# Protocol for measuring beta-secretase activity in cell and tissue lysate using a fluorogenic peptide substrate in a 96-well format

**DOI:** 10.1016/j.xpro.2025.103885

**Published:** 2025-06-07

**Authors:** Ahmad Mohammad, Emily N. Copeland, Val A. Fajardo, Rebecca E.K. MacPherson

**Affiliations:** 1 Department of Health Sciences, Brock University, St. Catherines, ON, Canada; 2 Department of Kinesiology, Brock University, St. Catherines, ON, Canada; 3 Centre for Neuroscience, Brock University, St. Catherines, ON, Canada

**Keywords:** Cell culture, Health Sciences, Neuroscience

## Abstract

The protease β-secretase (BACE1) plays a crucial role in the formation of amyloid-beta peptides. Here, we present a protocol for real-time quantification of BACE1 activity in brain tissue and cell lysates using a fluorogenic peptide substrate in a 96-well format. We describe steps for reagent and sample preparation, preparing the plate for BACE1 activity, and incubating and reading the plate, followed by quantification and analysis. This protocol is broadly accessible for laboratories studying enzymatic activity under physiological or pathological conditions.

For complete details on the use and execution of this protocol, please refer to Baranowski et al.^1^

## Highlights

• Steps for quantifying BACE1 activity using a fluorogenic peptide substrate
• Instructions for real-time endpoint and kinetic measurements in a 96-well plate format
• Requires minimal sample volumes
• Guidance on adaptations to various tissues and cell lysates

## Abstract (editor highlights)

Publisher’s note: Undertaking any experimental protocol requires adherence to local institutional guidelines for laboratory safety and ethics.

## Graphical abstract

**Figure:**
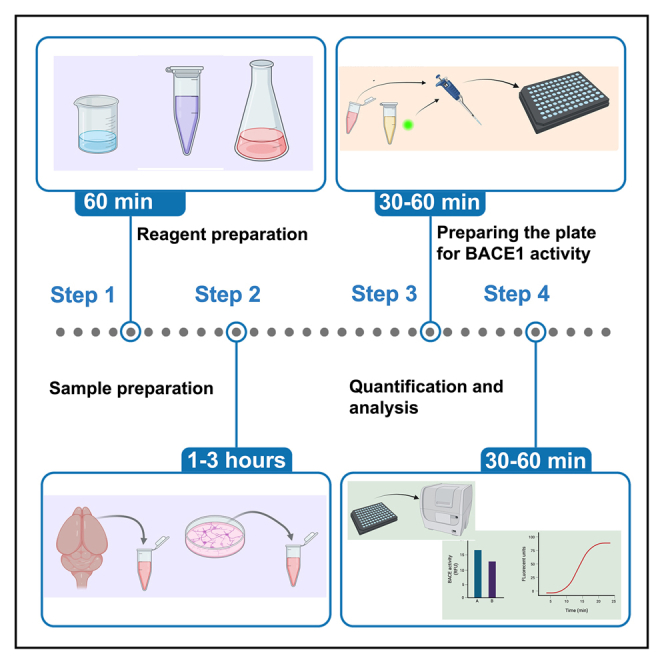

## Before you begin

**Figure 1:**
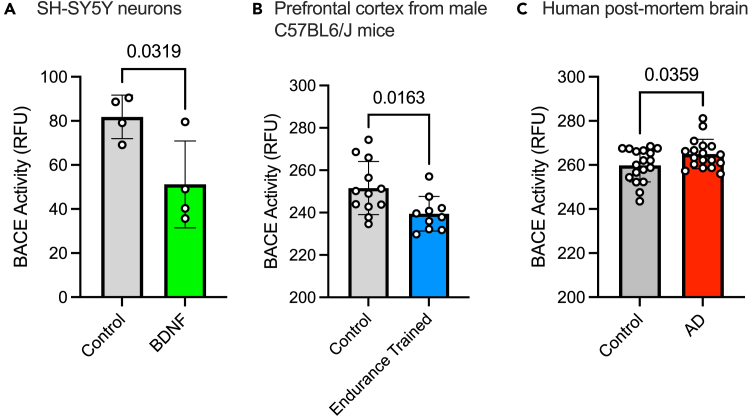
BACE activity obtained in SH-SY5Y cell lysates and mouse brain and human brain tissue lysates (A) Thirty minute incubation of differentiated SH-SY5Y neurons with BDNF (0.75 ng/ml) results in lower BACE activity. (B) Eight weeks of treadmill training results in lower prefrontal cortex BACE activity in male C57BL6/J mice. (C) Post-mortem brain samples from patients with Alzheimer’s disease have higher BACE activity. Values are means +/−SD.

The following protocol describes the steps for measuring β-secretase (BACE1) activity in murine and human brain sample homogenates as well as SH-SY5Y differentiated neurons. Notably, we have been able to successfully use these protocols in mouse and human brains although it can be adapted to measure β-secretase activity in different tissues as needed (Figure 1).

### Institutional permissions (if applicable)

All animal experiments were in compliance with the Brock University Animal Care Committee and the Canadian Council on Animal Care. All human experiments were in compliance with the Brock University Research Ethics Board. Researchers will need to check and/or obtain permissions from the relevant institutions before conducting this protocol.

### Reagent preparation for BACE1

**Timing: 60 min**

1. Prepare sample homogenizing buffer:
  a. Combine 10 ml of cell lysis buffer (Invitrogen FNN0021) with 34 μL of protease inhibitor (Sigma P7626-56) and 50 μL of phosphatase inhibitor (Sigma P2714-1).
2. Prepare Assay Buffer (0.2 M Sodium Acetate).
  a. Dissolve 16.4 g of anhydrous sodium acetate (NaC2H3O2) or 27.2 g of sodium acetate trihydrate (NaC2H3O2·3H2O) in 800 ml of dH_2_O, bring to a pH of 4.5 using acetic acid, fill to 1 L using dH_2_O.
3. β-secretase substrate (250 μM, Sigma 565758).
  a. Dissolve 1 mg into 2 mL of DMSO. Prepare 100 μL aliquots and store at −20°C. Stock solutions are stable for up to 1 month at −20°C. This substrate is light sensitive therefore aliquots should be placed in opaque tubes or tubes wrapped in tin foil.
4. β-secretase inhibitor (250 μM, Sigma S4562).
  a. Dissolve 1 mg into 2.42 mL DMSO. Prepare 100 μL aliquots and store at −20°C. Stock solutions are stable for up to 1 month at −20°C.

### Tissue preparation

**Timing: 1–3 h (timing will depend on number of samples)**

5. Tissue Preparation.
  a. Following the dissection of the desired brain region or tissue, snap freeze samples in liquid nitrogen and store them at −80°C.
  b. Homogenize brain tissue with a 1 mg: 20 μL ratio with homogenization buffer (e.g., 5 mg of sample will be homogenized in 100 μL of buffer).
  c. Homogenize samples using either a FastPrep homogenizer (Savant Bio 101 FastPrep FP101 Cell disruption system) set to the appropriate speed and duration (i.e. 6.5 and 45 s is appropriate for most preparations) or homogenize by hand using glass conical homogenizers.
  d. Let homogenates rest in fridge at 4°C for 20 min.
  e. Centrifuge homogenates at 10,000 RCF for 15 min at 4°C (specific for brain tissue homogenates).
  f. Carefully collect the supernatants from each sample, making sure to not disturb the pellet, and transfer each to its own separate Eppendorf tube.
  g. Quantify protein concentration in each sample (Bicinchoninic acid assay or Bradford assay). Obtain concentration in μg/μL.

***Note:*** Samples can be frozen and stored at −80°C prior to and/or after the protein concentration is determine and the activity assay can be performed at a later date.

***Note:*** We have also successfully performed this activity assay on SH-SY5Y cells. Collect cells using the same lysis buffer as tissue homogenization. The ratio of lysis buffer to cells is sample-dependent (adjust based on plate size to optimize protein concentration, for example in a 6 well plate lyse cells with 200 μL lysis buffer per well).

After homogenization, sonicate samples 3 × 15 s (with cooling intervals) to ensure homogeneity before quantifying protein concentrations.

## Key resources table

**Table:** 

REAGENT or RESOURCE	SOURCE	IDENTIFIER
**Chemicals, peptides, and recombinant proteins**		

Cell lysis buffer	Thermo Fisher Scientific, Waltham, MA	FNN0021
Phenylmethanesulfonyl fluoride (PMSF)	Sigma–Aldrich, Oakville, ON	P7626
Protease inhibitor	Sigma–Aldrich, Oakville, ON	P2714
β-secretase substrate IV, fluorogenic	Sigma–Aldrich, Oakville, ON	565758
β-secretase inhibitor	Sigma–Aldrich, Oakville, ON	S4562
Active β-secretase	Sigma–Aldrich, Oakville, ON	S4195
Sodium acetate	Sigma–Aldrich, Oakville, ON	S2889

**Software and algorithms**		

SoftMax Pro 7 software	Molecular Devices	N/A

**Biological samples**		

Human postmortem brain homogenate, male (78.6 ± 8.1 years of age) and female (79.4±7.8 years of age) non-demented controls with no AD diagnosis, and male (82.4±5.9 years) and female (81.2±7.0 years of age) samples with AD status	The Douglas Bell Canada Brain Bank (Montreal, Quebec, Canada; http://douglasbrainbank.ca)	N/A

**Experimental models: Cell lines**		

SH-SY5Y neuroblastoma cell line	ATCC	N/A

**Experimental models: Organisms/strains**		

Murine (male, C57BL/6, 3–4 months) Prefrontal cortex homogenate	The Jackson Laboratory	N/A

**Other**		

All black 96-well plates	Thermo Fisher Scientific, Waltham, MA	7805
Eppendorf repeater pipette	Sigma–Aldrich, Oakville, ON	EP4982000322
SpectraMax M2 microplate reader	Molecular Devices, San Jose, CA	https://www.moleculardevices.com/products/microplate-readers/
1.5 mL microcentrifuge tubes	Thermo Fisher Scientific, Waltham, MA	14-222-158

## Step-by-step method details

### Preparing a plate for BACE activity

**Timing: 30 min to 1 h**

This step is for plating the necessary components of the BACE activity assay prior to kinetic or endpoint readings.

1. Set up reaction wells.
  a. Background well = 50 μL assay buffer.
  b. Positive control well = 46 μL assay buffer + 4 μL active β-secretase.
  c. Negative control well = 44 μL assay buffer + 4 μL active β-secretase + 2 μL β-secretase inhibitor.
  d. Sample wells = using dH_2_O dilute all samples to an equal concentration (0.5–2 μg/ μL). The total volume of samples must be at least 120 μL.
2. Load 50 μL of sample into each well in duplicate.
3. Add 48 μL of Assay buffer to each well (including control wells) using a multichannel pipette.
4. In a dark room, add 2 μL of β-secretase substrate to each well. This substrate is sensitive to light, so loading should be performed under low light conditions. For light-sensitive substrates, a common recommendation is to use red light at or below 50 lux.
5. Cover the plate with a layer of aluminum foil and tap the sides gently to mix the solutions.

### Incubate and read the plate

**Timing: 30 min to 1 h**

This step guides through the endpoint and kinetic readings of the prepared plate.

**Figure 2:**
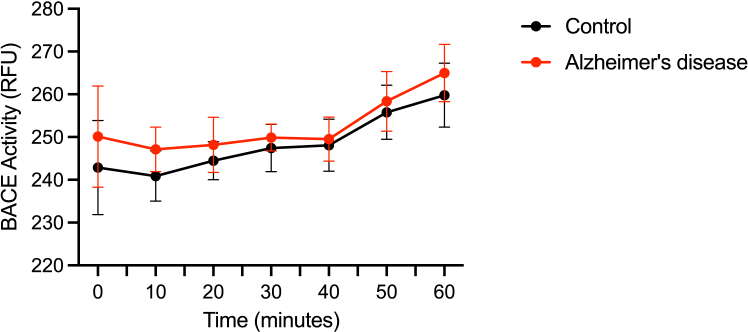
BACE activity obtained in post-mortem brain tissue lysates over 60 min kinetic assay Black line and data points represent control non-demented samples. Red line and data points represent Alzheimer’s disease samples. Sample size of 18 for each group and each time point. Values are means +/−SD.

6. Incubate and read plate.
  a. For endpoint assay: Incubate at 37°C for 30 min in dark room. Measure output on a fluorescent microplate reader set to fluorescent endpoint reading 96-well opaque plate at Ex/Em = 350/490 nm.
  b. For kinetic assay: Set fluorescent microplate reader to 37°C and ensure that it is set to a fluorescent kinetic setting reading an opaque 96 well plate. Set the total read time to 60 min, with intervals reading fluorescence every 5 min at Ex/Em = 350/ 490 nm (Figure 2).

***Optional:*** If you intend on determining IU/mg protein rather than RFU for the assay then a standard curve is required.

7. Set up reaction wells.
  a. Background well = 50 μL assay buffer.
  b. 48 μL assay buffer + 2 μL β-secretase.
  c. 46 μL assay buffer + 4 μL β-secretase.
  d. 42 μL assay buffer + 8 μL β-secretase.
  e. 34 μL assay buffer + 16 μL β-secretase.
  f. 32 μL assay buffer + 16 μL β-secretase + 2 μL β-secretase inhibitor.
  g. Sample wells = using dH_2_O dilute all samples to an equal concentration (0.5–2 μg/ μL). Samples must be at least 120 μL.
8. Load 50 μL of sample into each well in duplicate.
9. Add 48 μL of Assay buffer to each well (including control wells) using multichannel pipette.
10. In a dark room, add 2 μL of β-secretase substrate to each well. This substrate is sensitive to light so loading should be performed using only red light.
11. Cover plate with layer of aluminum foil and tap sides gently to mix.
12. Incubate and read plate.
  a. For endpoint assay: Incubate at 37°C for 30 min in dark room. Measure output on a fluorescent microplate reader set to fluorescent endpoint reading 96-well opaque plate at Ex/Em = 350/490 nm.
  b. For kinetic assay: Set fluorescent microplate reader to 37°C and ensure that is set to a fluorescent kinetic setting reading an opaque 96 well plate. Set total read time to 60 min with intervals reading fluorescence every 5 min at Ex/Em = 350/ 490 nm.
13. Export all data to Excel.
14. The standard curve of 2, 4, 8, 16 μL would be equal to 1 μg, 2 μg, 4 μg and 8 μg = 20, 40, 80, and 160 units of activity. Use this data with the standard curve to determine the units of activity within each biological sample.

## Expected outcomes

When successfully implemented, this protocol enables the detection and quantification of BACE1 proteolytic activity through fluorescent signal measurement. The assay serves two primary functions: it evaluates changes in BACE1 activity in diverse models, including in vivo systems and cell culture environments, and it facilitates high-throughput screening for novel BACE1 inhibitors. This makes it a valuable tool for both experimental analysis and drug discovery, providing insights into BACE1 activity and aiding in the identification of potential therapeutic agents.

## Quantification and statistical analysis

1. Data Preparation.
  a. Transfer all raw data to a spreadsheet format (e.g., Excel/Google Sheets).
2. Assay Quality Control.
  a. Validate assay performance by analyzing control wells:
    i. **Positive controls**: Should yield the highest values.
    ii. **Background/Negative controls**: Expected to show comparable baseline values.
    iii. *Proceed only if controls meet these QC criteria.*
3. Data Processing.
  a. Calculate the mean of technical replicates (duplicate samples).
  b. Normalize data as needed and group samples by experimental condition.
  c. Report values in relative fluorescent units (RFU).
4. Statistical Analysis.
  a. Use appropriate tests based on experimental design:
    i. Parametric tests (e.g., Student’s *t*-test, ANOVA) for normally distributed data.
    ii. Non-parametric alternatives (e.g., Mann-Whitney *U*, Kruskal-Wallis) for non-normal distributions.
  b. Specify statistical methods, significance thresholds (e.g., *p* < 0.05), and software used (e.g., GraphPad Prism, R).

## Limitations

The assay’s narrow specificity, mimicking only the Swedish mutant APP cleavage site, may overlook BACE1 interactions with other physiological substrates, limiting insights into off-target effects on non-APP pathways.

## Troubleshooting

### Problem 1

Samples cannot be prepared to the desired range of concentrations (0.5–2 μg/ μL) (Preparing a plate for BACE activity step 6d).

### Potential solution

• If sample is too concentrated (>2 μg/μL) continue to dilute the samples and consider serial dilution steps.
• If the sample is too dilute (<0.5 μg/μL) use a larger sample volume or concentrate the sample via evaporation or freeze-drying.
• If the desired range cannot be attained, adjust the assay to include a wider range of standards.

### Problem 2

No fluorescent signal is detected upon the addition of the enzyme to the substrate solution during the measurement of the enzymatic activity. (Incubate and read the plate step 11).

### Potential solution

• Consider using a new β-secretase substrate solution, as it is sensitive to light and may degrade over time.
• Increase the concentration of the β-secretase substrate in the mixture. The amount of substrate can be adjusted based on experimental needs. Optimization typically involves testing a range of substrate concentrations to determine the optimal amount that provides a robust signal without reaching substrate saturation.
• Extend the reaction monitoring period. Endpoint assays can be measured at 30 and 60 min.
• It may be beneficial to test varying concentrations of both the sample and the substrate initially to evaluate the sensitivity of your instrument.

### Problem 3

Reagent stability issues in the assay, particularly the degradation of critical components like peptide substrates and enzymes due to repeated freeze-thaw cycles.

### Potential solution

• Implement a single-use aliquot system for reagents.
• Prepare smaller volumes of peptide substrates and enzymes and store them in individual aliquots that can be thawed only once before use
• This approach minimizes freeze-thaw cycles, preserves reagent integrity, and enhances assay reproducibility.
• Consider using stabilizing agents or lyophilization techniques for long-term storage of sensitive components.

## Resource availability

### Lead contact

Further information and requests for resources and reagents should be directed to and will be fulfilled by the lead contact, Rebecca EK MacPherson (rmacpherson@brocku.ca).

### Technical contact

Val A Fajardo (vfajardo@brocku.ca) and Rebecca EK MacPherson (rmacpherson@brocku.ca).

### Materials availability

This study did not generate new unique reagents.

### Data and code availability

This study did not generate/analyze [datasets/code].

## Acknowledgments

This work was supported by an NSERC Discovery Grant to R.E.K.M. V.A.F. is supported by a Canada Research Chair (Tier 2) award. A.M. is supported by an NSERC CGS-M award. E.N.C. is supported by an NSERC CGS-D award.

## Author contributions

A.M. and E.N.C. helped conceive the protocols; optimized the protocols; performed data collection and analysis; and wrote, reviewed, and edited the manuscript. V.A.F. and R.E.K.M. conceived the protocols, helped optimize the protocols, and reviewed and edited the manuscript.

## Declaration of interests

The authors declare no competing interests.
